# P-1109. Steroid Duration and Mucormycosis Severity in COVID-19: A Dose-Response Relationship

**DOI:** 10.1093/ofid/ofaf695.1304

**Published:** 2026-01-11

**Authors:** Darshankumar Raval, Masum S Patel, Shubham N Patel, Ahan Banker, Vaishnavi M Rathod, Justin Oring, Munir P Shah, Virginia D Banks

**Affiliations:** Mayo Clinic, Jacksonville, Youngstown, Ohio; B.J.MEDICAL COLLEGE, AHMEDBAD, Mehsana, Gujarat, India; B.J.MEDICAL COLLEGE, AHMEDBAD, Mehsana, Gujarat, India; B.J. Medical College and Civil Hospital, Ahmedabad, Ahmedabad, Gujarat, India; One Brooklyn Health Interfaith Medical Center, Brooklyn, New York City, NY; Mayo Clinic Florida, Jacksonville, Florida; Western Reserve Health Education/ NEOMED, Sharon, Pennsylvania; Northeast Ohio Infectious Disease Associates, Youngstown, OH

## Abstract

**Background:**

The COVID-19 pandemic saw a worrisome rise in mucormycosis cases in India, especially among those receiving corticosteroid therapy. While corticosteroids are essential for managing severe COVID-19 cases, their immunosuppressive action appears to augment infection risk. Clinically, prolonged steroid use may result in severe forms of mucormycosis; however, this requires rigorous systematic evaluation.

**Methods:**

We conducted an exhaustive analysis of 327 mucormycosis cases and 596 COVID-19 controls, all of whom were treated during the second wave in India. Exposure to steroids was carefully categorized by duration into three groups: short-term (1-5 days), standard-term (5-10 days), and long-term ( > 10 days). Each case was biopsy-proven, with further radiographic confirmation of disease extent. Special emphasis was laid on intracranial extension as a marker of severity, along with tracking treatment outcomes and survival. Statistical modeling was used to correct for important confounders, including diabetes status, glycemic control, and concurrent use of other immunomodulators.

**Results:**

The results were striking. Those receiving steroid treatment for more than ten days of duration had a nearly fourfold higher incidence of intracranial mucormycosis compared with short-term-treated patients (38.2% vs. 12.5%, p< 0.001). Each day of steroid use accounted for a 12% increase in associated risk. While mortality rates were equal, patients receiving prolonged treatment had a considerably increased number of surgical procedures (35.3% vs. 16.7%, p=0.02) as well as more complicated hospitalization. Those with diabetes were especially sensitive to such effects of prolonged treatment.

**Conclusion:**

The study highlights the role of cautious use of corticosteroids in the management of COVID-19. Our work gives strong evidence for the importance of compliance with guideline-based recommendations for corticosteroid duration, especially for high-risk populations. This study empowers healthcare practitioners with unequivocal evidence to provide optimal COVID-19 treatments while avoiding the risk of severe fungal infection.

**Disclosures:**

All Authors: No reported disclosures

